# Near-Room-Temperature Detection of Aromatic Compounds with Inkjet-Printed Plasticized Polymer Composites

**DOI:** 10.1021/acssensors.3c02406

**Published:** 2024-03-13

**Authors:** Mohammad Mahdi Kiaee, Tomas Maeder, Juergen Brugger

**Affiliations:** Microsystem Laboratory, École Polytechnique Fédérale de Lausanne (EPFL), CH-1015 Lausanne, Switzerland

**Keywords:** gas sensor, volatile organic compounds, polymer composite, inkjet printing, plasticizer, carbon black, design of experiment

## Abstract

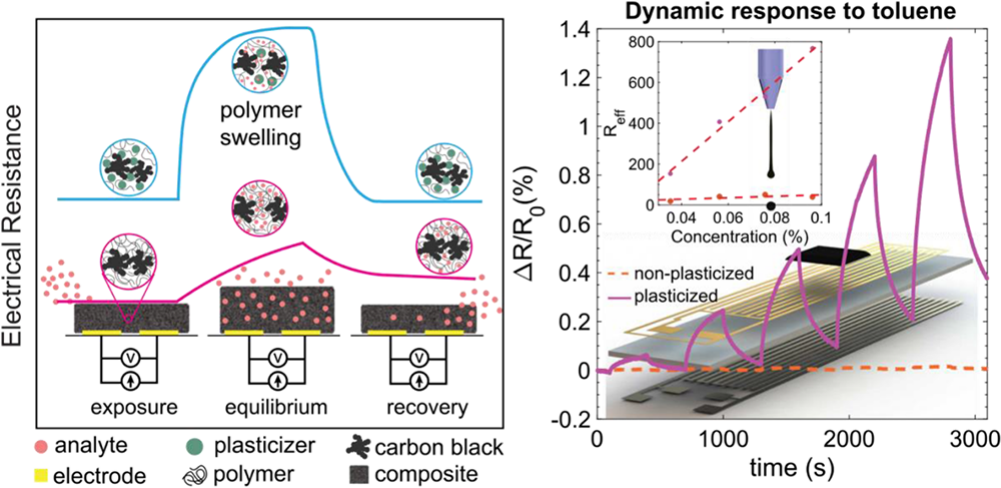

Chemiresistive gas sensors composed of a thermoplastic polymer matrix and conductive fillers offer various advantages for detecting volatile organic compounds (VOCs), including low power consumption due to near-room-temperature operation, high sensitivity, and inherent selectivity toward VOCs. However, such sensors have a slow response time as the polymer matrix often has a glass transition temperature (*T*_g_) higher than the sensor operating temperature slowing the analyte diffusion to/from the polymer. A plasticizer lowers polymer *T*_g_ to match the sensor operation temperature, reducing its response time. In this study, the effect of a plasticizer diethylene glycol dibenzoate (DEGDB) on the sensing properties of polystyrene (PS)-carbon black (CB) composite is investigated to obtain sensors with a fast response time and high sensitivity to VOCs. The sensors are fabricated via drop-on-demand inkjet printing, providing a high degree of control over the sensory film morphology and reproducibility. A design-of-experiment (DoE) approach is adopted to find the optimum ink and print parameters with a minimum number of experiments. As a result, sensors with 30 times faster response time and 25 times higher effective sensitivity are obtained while operating near room temperature (27 °C). Furthermore, the sensors show high sensitivity toward aromatic hydrocarbons (toluene, benzene, and ethylbenzene), with a sub-10 ppm limit of detection (LoD) and a negligible sensitivity toward humidity. Our results show the potential of PS-DEGDB-CB composite as a selective and cost-effective sensory material compatible with large-scale manufacturing techniques for selective near-room-temperature detection of toxic VOCs.

Detecting trace amounts of volatile organic compounds (VOCs) has important applications in environmental and health monitoring domains, be it by detecting exogenous VOCs that can pose health risks,^1^ or detecting endogenous, i.e., bodily VOCs, that act as biomarkers.^2^ A simple yet effective way to detect VOCs is by utilizing detectors whose resistance varies upon exposure to the target analyte, so-called chemiresistive sensors. Such sensors have been studied extensively over the past few decades using a wide range of materials owing to their relatively simple architecture, fabrication, and characterization process. The main objectives of most research studies include improving the sensor sensitivity, selectivity, repeatability, and LoD while minimizing the power consumption for wearables and Internet-of-things (IoT) applications, extending the sensor lifetime, and employing scalable manufacturing processes.^3−5^

Sensors with a detecting element composed of a polymer matrix and conductive fillers, i.e., polymer nanocomposites (PNCs), are suitable candidates to address the various requirements mentioned above. PNCs are highly sensitive and inherently selective toward VOCs, determined by their solubility parameters.^6^ Moreover, sub-ppm LoD can be obtained in such sensors, depending on the analyte’s vapor pressure, while operating at near room temperature with low power consumption.^7^ Additionally, PNC-based chemiresistors are compatible with scalable fabrication methods such as inkjet printing.^8^

Despite their various advantages, detectors composed of a thermoplastic polymer matrix display kinetics of the sensor response that is highly dependent on the diffusivity of the analyte molecules to the polymer matrix. The diffusivity is primarily determined by the glass transition temperature (*T*_g_) of the polymer matrix.^9^ Therefore, in sensing materials composed of a thermoplastic with a relatively high *T*_g_, such as PS, the response and recovery are very slow, hindering the sensor performance and limiting its applications.

Various methods can circumvent the diffusion limitation in high *T*_g_ polymers. For example, the application of thin-film PNC improves the sensor response time; however, as the film thickness becomes comparable to the conductive particle size, the sensor’s baseline noise increases.^10^ On the other hand, increasing the sensor temperature to above the polymer *T*_g_ improves the analyte’s diffusivity. However, it increases the power consumption and lowers the sensor sensitivity, due to the increased rate of analyte desorption at elevated temperatures.^11^ An alternative option is to lower the polymer *T*_g_ using a plasticizer, allowing faster absorption and desorption of analytes to and from the polymer matrix at room temperature. Koscho et al. have demonstrated the application of plasticized poly(vinyl acetate) and poly(*N*-vinylpyrrolidone) for producing chemically different detectors in sensor arrays.^12^ They have demonstrated that a rapid sensor response can be obtained at room temperature while the sensor sensitivity is altered by introducing the plasticizer.

Following Koscho et al.’s study, we have systematically investigated how plasticizing a composite containing PS and CB, using DEGDB, can improve the sensor’s performance. The expected dynamic response of a nonplasticized versus a plasticized PS-CB composite is shown in Figure 1a to illustrate the improvement of the sensor response resulting from plasticizing the polymer. The figure demonstrates that a nonplasticized composite shows a slow response due to the slow analyte diffusion. When the polymer is in its glassy state, the analyte has first to soften the polymer, which slows its diffusion into the polymer. Moreover, the glassy nature of the polymer results in slow and incomplete sensor recovery after the removal of the analyte. On the other hand, adding a plasticizer and lowering the polymer *T*_g_ enhances the mobility of polymer chains and improves the analyte diffusion resulting in a fast equilibration of the sensor response followed by a rapid and complete recovery.

**Figure 1:**
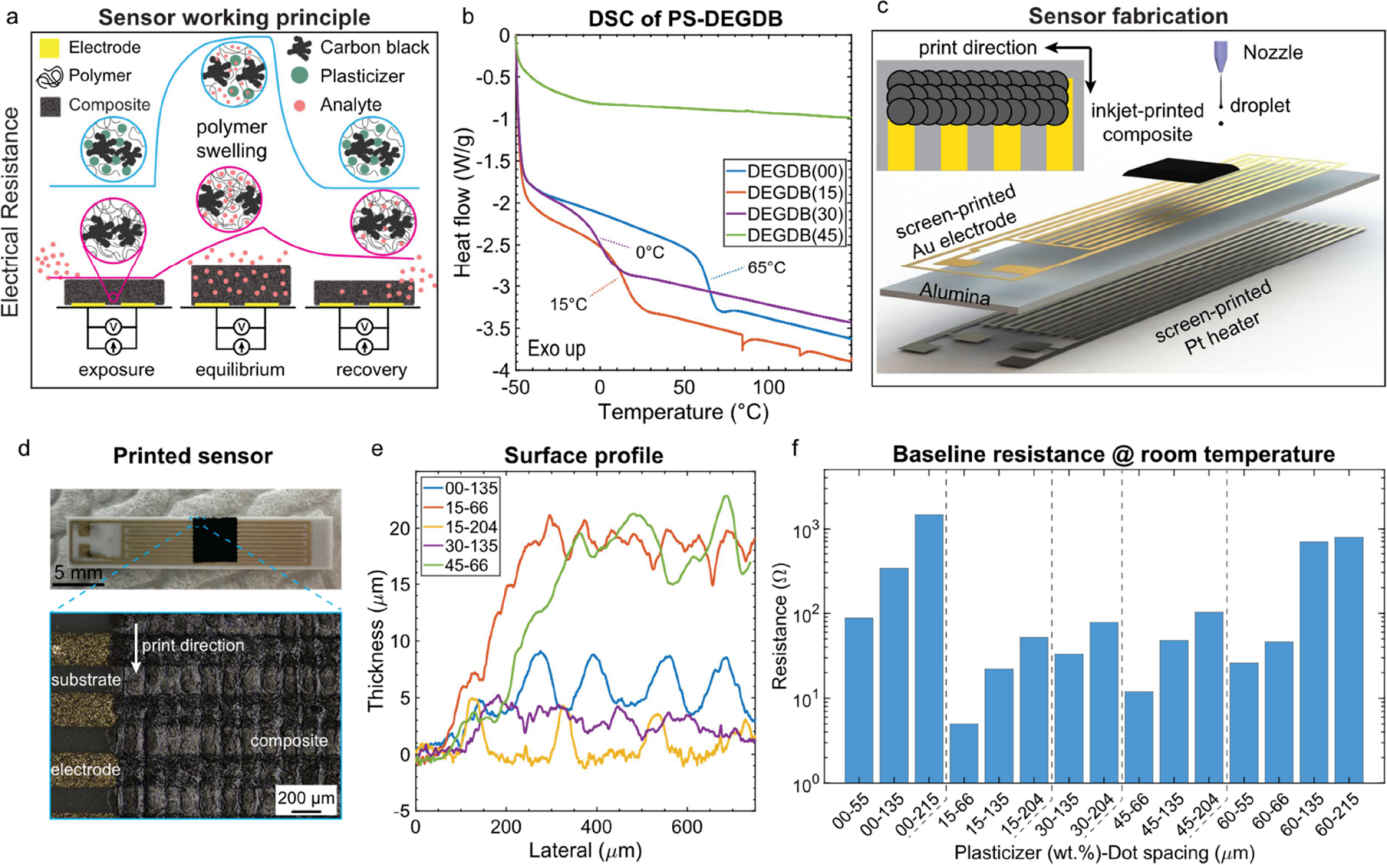
(a) A schematic illustration of the plasticizing effect on the sensing performance of a PS-CB composite. In the absence of a plasticizer, the sensor response is slow, with a partial recovery (red curve). In the presence of a plasticizer, the sensor responds rapidly to the analyte and is fully recovered after the analyte is removed (blue curve). (b) DSC measured from −50 to 150 °C with a heating rate of 10 °C/min. The figure illustrates the decrease of PS *T*_g_ by increasing the DEGDB weight fraction from 65 to 0 °C at 30 wt % DEGDB. (c) Schematic illustration of an inkjet-printed sensory film on an alumina substrate with screen-printed Au IDEs and a Pt heater. The inset shows how the sensory film is formed from the coalescence of the neighboring droplets. (d) A photograph (top) of a 4 × 4 mm^2^ sensory film. The sensory film is composed of PS, DEGDB, and CB, printed with 135 μm dost spacing and 5 repeat times. The bottom image shows a micrograph of the sensing layer. (e) The surface profiles of the sensory films measured with a Dektak mechanical profilometer. It is observed that at a constant plasticizer concentration, increasing the dot spacing reduces the film thickness. On the other hand, Increasing the DEGDB concentration reduces the film thickness at a fixed dot spacing. (f) The room temperature baseline resistance of the printed sensors. At a constant plasticizer concentration, increasing the dot spacing increases the baseline resistance. At a constant dot-spacing (e.g., 135 μm), adding the plasticizer (from 0 to 15 wt % DEGDB) reduces the baseline resistance by approximately 1 order of magnitude. However, increasing the plasticizer concentration further (15–60 wt %) increases the baseline resistance gradually.

The PS-CB system is particularly of interest since based on its constituents’ HSPs,^13^ it is expected to be highly sensitive toward carcinogenic compounds such as benzene, ethylbenzene, toluene, and xylene, so-called BTEX compounds. To be compatible with advanced micromanufacturing, we have formulated inks containing PS-CB-plasticizer with tailored properties for drop-on-demand inkjet printing and investigated the effect of the ink composition and print parameters on the sensor performance. As a result, we demonstrated here that low-power, cost-effective sensors with reproducible performance and high selectivity toward BTEX compounds can be obtained by optimizing the ink composition, i.e., plasticizer concentration and print parameters.

## Experimental Section

### Material Selection and Ink Formulation

The polymer used in this study is PS with a molecular weight of 35 kg/mol (https://www.sigmaaldrich.com/product/aldrich/331651) as indicated by the product specification sheet. A high-structure CB, i.e., Ketjenblack EC-600JD (Nouryon), is used as the conductive filler. Application of a high structure CB allows obtaining conductive polymer composites at relatively low CB loadings (∼6 vol %).^14^ The PS polymer matrix is plasticized using DEGDB supplied by TCI chemicals (purity >97.0%). Inkjet inks are prepared using a solvent mixture containing propylene glycol methyl ether acetate (PGMEA) and dipropylene glycol methyl ether acetate (DPGMEA). Both solvents are reagent grade and were purchased from Sigma-Aldrich.

The inkjet ink formulation was performed as follows: PS, CB, and DEGDB were mixed in glass vials in different weight ratios. Subsequently, PGMEA and DPGMEA were added to the mixture in a 7:3 weight ratio. The sensing materials (i.e., PS, DEGDB, and CB) comprised 12 wt % of the total ink mass for optimal printability. After the mixture was prepared, PS was dissolved in the solvent by magnetically stirring the ink. Subsequently, the inks were sonicated at 150 W for 5 min to disperse the CB particles. The inkjet inks were then centrifuged at 13 krpm for 1 min to sediment large CB aggregates that could potentially clog the nozzle. The supernatant was then used for printing.

It should be noted that the parameters used for the ink formulation were based on our previous study on the printability of PS-CB composites.^15^ Therefore, the base formulation from our previous paper was used in the current study; however, to adjust for the ink formulation when adding the plasticizer, a particular mass fraction of PS was replaced by DEGDB considering the target PS-DEGDB composition.

Two sets of inks were prepared. The first set contained a fixed amount of CB (10 wt % of the total sensing material, i.e., the sum of PS, CB, and DEGDB) and different polymer/plasticizer ratios. The aim was to find the optimum DEGDB concentration in ink that would result in optimum sensor functionality. In these inks, the sum of the PS and DEGDB weight fractions remained constant, and adding more plasticizers meant replacing a given PS mass by DEGDB. Consequently, inks containing 0, 15, 30, 45, and 60 wt % DEGDB with respect to PS content were prepared. The compositions of the dry composites containing PS, DEGDB, and CB are shown in Table S1 in terms of both the volume and weight fractions.

The second set of inks was prepared with a fixed DEGDB:PS ratio but different CB contents. The aim was to study the effect of CB concentration on the sensor functionality. Hence, inks containing 10, 8, 6, 4, and 2 wt % CB in dry composites were formulated. Table S2 shows the detailed compositions of dry composites with different CB loading.

### Sensor Fabrication

The sensing platform comprises an alumina substrate with screen-printed Au interdigitated electrodes (IDEs) and Pt heaters. The line width and spacing between the IDE and heater fingers are both 200 μm. Even though the sensing material can operate at room temperature, adding a heating element on the substrate allows heating of the sensor slightly above room temperature and thus avoids the impact of environmental temperature fluctuations on the sensing performance.

The sensor platforms were cleaned with various solvents before printing the sensing material. The cleaning was performed by successive immersion of the substrates in acetone, 2-propanol, and deionized (DI) water in a sonication bath for 5 min for each solvent. Subsequently, the substrates were dried on a hot plate at 100 °C for 1 h before printing the sensing material.

The sensory films were patterned using a drop-on-demand inkjet printer equipped with an 80 μm nozzle on an area of 4 × 4 mm^2^ at the center of the sensing platform (using a customized MicroFab inkjet printer). The sensory films were printed while the substrate temperature was kept at 60 °C, the wait time between two droplet bursts was set to 100 ms, and the dot spacing varied from 55 to 215 μm. Furthermore, the print repeat time was set to 5, meaning that 5 layers of the composite were printed at a fixed dot spacing to fabricate each sensor. After printing, the sensory films were dried at 90 °C for 1 h before the characterizations.

### Material and Sensor Characterization

Glass transition temperature and thermal stability of PS and DEGDB were measured using a TA Instrument DSC Q100 and TGA 4000 instruments from PerkinElmer. Following the printing of the sensory films, their room-temperature electrical resistance was measured with a digital multimeter under ambient conditions. Subsequently, the film’s thickness was measured using a Bruker Dektak mechanical profilometer equipped with a 12.5 μm stylus. The printed films’ morphology and microstructure were studied using a Nikon optical microscope and Zeiss Merlin scanning electron microscope (SEM).

The VOC sensing performance was characterized upon exposure to calibrated concentrations of various analytes. A detailed description of the characterization setup can be found in our previous publication.^14^ In summary, a controlled flow of dry air was passed through a bubbler containing solvents of interest. The bubbler temperature was controlled by using a water-cooled bath maintained at 16 °C. The saturated analyte flow exiting the bubbler was mixed with a background flow of dry air (2000 mL/min). The diluted analyte flow was then guided toward the sensor chamber.

The sensor temperature was kept constant at 27 °C via integrated heaters on sensor platforms in all measurements to avoid the parasitic effect of ambient temperature variations. The sensor resistance changes upon analyte exposure were monitored with a Keithley 2000 digital multimeter. The dynamic response of each sensor was recorded using a LabView program, from which the sensors’ features, including response magnitude, response time, and baseline noise, were extracted.

Two sets of sensing measurements were carried out. First, the printed sensors with different DEGDB concentrations and dot spacings were exposed to a constant acetone concentration (∼0.4% vol/vol). Each sensor was exposed to acetone for 5 min and 5 min recovery under dry air. The same protocol was used to characterize sensors with different CB loadings. These measurements led to finding the best-performing sensor printed with the optimum dot spacing and composition.

The second set of experiments was performed to study the selectivity and sensitivity. Therefore, the best-performing sensor was exposed to various analytes, including water, ethanol, 2-propanol, acetone, heptane, benzene, ethylbenzene, and toluene. Moreover, the concentration dependence of the sensor response was investigated, where the concentration gradually increased by increasing the dry airflow passing the bubbler.

## Results and Discussion

### Material Characterization

The effect of the plasticizer on the glass transition temperature of PS was investigated by conducting DSC on samples containing 0, 15, 30, and 45 wt % DEGDB. The first DSC cycle was performed to remove the polymer history effect, by heating the samples from 20 to 150 °C and then cooling to −50 °C with a heating rate of 10 °C/min. Subsequently, the second DSC cycle was performed from −50 to 150 °C for measuring the *T*_g_. The heat flow curves illustrating PS *T*_g_ as a function of DEGBD concentration are shown in Figure 1b. The as-received PS shows a *T*_g_ of 65 °C, which is lower than the 95 °C that is reported in the literature. A possible explanation for this discrepancy could be linked to a high degree of polydispersity and low molecular weight species present in PS. The weight-average molecular weight reported in the material data sheet (35 kg/mol) is generally biased toward high molecular weights. Depending on the polydispersity of the polymer, the number-average molecular weight could be much smaller than this value; hence, the presence of short PS chains results in a lower glass transition temperature.^16^

The PS *T*_g_ is decreased significantly by adding 15 and 30 wt %, DEGDB to 15 and 0 °C, respectively. At 45 wt % DEGDB, the DSC curve does not show a glass transition, indicating that the *T*_g_ drops below −50 °C, the minimum temperature that can be reached with our measurement tool under the cooling rate of 10 °C. Nevertheless, lowering the PS *T*_g_ to below room temperature by adding more than 15 wt % DEGDB is expected to improve the sensor kinetics primarily by increasing the free volume between polymer chains, allowing the chains to move more freely, and promoting a faster diffusion of the analyte to/from the polymer.

### Design of Experiment

A DoE approach based on the uniform shell design, i.e., the Doehlert method,^17^ is employed to optimize the plasticizer concentration and the printing parameter, i.e., dot spacing. The Doehlert method allows studying the effect of the two continuous factors by performing 7 experiments. Furthermore, the experimental points generated by the Doehlert method result in combinations of factors that are equidistant from the center of the DoE space, allowing investigation of the effect of two or more factors at the same time.

Following the DSC measurement, the range for DEGDB concentration is considered between 0 and 60 wt %. The dot spacing ranges from 55 to 215 μm, corresponding to a 20–80% overlap between the neighboring droplets. The overlap is calculated based on the diameter of a single droplet on the substrate, approximately 270 μm for the nonplasticized ink.

In addition to the combinations of factors based on the Doehlert method, four sensors were fabricated with different combinations of minimum and maximum dot spacings and DEGDB concentrations. The additional sensors help predict the sensing behavior at the corners of the DoE space since the Doehlert design does not cover those areas. Table 1 shows the experimental matrix, including 11 sensors with different DEGDB-dot spacing combinations used to find the optimum sensor.

**Table 1:** Matrix of the Experiment That Was Obtained Based on the Doehlert DOE Method

DEGDB (wt %)	0	15	15	30	45	45	60	0	60	0	60
dot spacing (μm)	135	66	204	135	204	66	135	55	55	215	215
label	00–135	15–66	15–204	30–135	45–204	45–66	60–135	00–55	60–55	00–215	60–215

### Inkjet Printing

Figure 1c shows a schematic view of a sensor composed of an alumina substrate, a screen-printed IDE and heater, and the inkjet-printed sensory film. The inset of the image shows how the sensory film is formed by overlapping the neighboring droplets. The spacing between the neighboring drops is adjusted according to the DoE matrix while the printing area remains constant (4 × 4 mm^2^). The print repeat time is set to 5, so the sensory film is thick enough to avoid noisy baseline resistance.

A photograph and a micrograph of a representative sensor composed of PS-CB, printed with a 135 μm dot spacing, are shown in Figure 1d. The irregular wavy surface observed in the sensory film is formed due to the material accumulation along the printed lines, formed as a result of the coffee ring effect. Increasing the overlap between the drops reduces the period of the wavy pattern until, above a critical overlap value, the coffee ring effect is no longer observed. On the other hand, by increasing the dot spacing, i.e., decreasing the overlap, the period of the wavy structure increases until a critical overlap value, below which the droplets are dried individually, with coffee rings forming around individual drops as shown in Figure S2.

Figure 1e shows the surface profile of the sensory film measured with a mechanical profilometer. For better readability, only the profiles of the selected samples are shown in the figure. The surface profiles of the remaining sensors are shown in Figure S3. It is observed that, as expected, decreasing the dot spacing at a constant plasticizer concentration increases the film thickness, since the amount of deposited material per unit area increases. On the other hand, increasing the DEGDB concentration decreases the film thickness at a fixed dot spacing. This observation is presumably linked to more spreading of inks containing high DEGDB concentration due to their lower viscosity compared to inks with high PS concentrations.

Variation of the dot spacing also affects the film morphology. For example, when the dot spacing is larger than 200 μm, coffee rings become prominent around the individual drops, as seen in sensor 15-204. However, increasing the droplet overlaps results in coffee ring formation along individual lines and creates a wavy pattern, as seen in sensor 00-135. Increasing the overlap further minimized the coffee ring formation, e.g., sensor 00-55.

On the other hand, increasing the plasticizer content removes the coffee rings even at relatively large dot spacings. For instance, the coffee rings do not appear in sensory films with 45 wt % DEGDB. This observation is explained considering that the *T*_g_ of PS drops below room temperature at 45 wt % DEGDB concentration, resulting in the liquid-like behavior of the sensory film. Hence, the neighboring droplets can merge and form a uniform film, even after solvent evaporation.

Figure 1f shows the room-temperature baseline resistance of the sensors. It appears that depending on the dot spacing and plasticizer concentration, the resistance varies between 5 and 1500 Ω. At a constant plasticizer concentration, the sensor resistance scales with the film thickness, resistance is decreased by increasing the film thickness. However, adding 15 wt % DEGDB reduces the baseline resistance by approximately 1 order of magnitude at a fixed dot spacing. From this point, the resistance gradually increases by increasing the plasticizer concentration from 15 to 60 wt %.

The significant initial reduction of the baseline resistance at 15 wt % DEGDB is linked to the distribution and connectivity of the CB network inside the PS-DEGDB matrix. Adding a plasticizer to a CB-polymer blend lowers the average molecular weight and melt viscosity. Subsequently, the percolation threshold concentration of the composite is reduced, resulting in a lower electrical resistivity at a fixed CB concentration than in a nonplasticized composite.^18^

The gradual increase of the baseline resistance, at a fixed dot spacing, by further increasing the DEGDB concentration in the matrix from 15 to 60 wt % is likely linked to the corresponding change in composite microstructure, as seen in the SEM images shown in Figure S4. Up to 30 wt % DEGDB (Figure S4a–d, a fine and quite homogeneous dispersion of CB in PS-DEGDB is formed. However, at 45% DEGDB (Figure S4e), the dispersion becomes much coarser, and visible CB-free zones form, appearing as bright islands in the SEM image. Increasing the DEGDB concentration to 60% (Figure S4f) furthers this trend with the CB-rich agglomerates separated by large insulating zones.

### Print and Plasticizer Optimization

**Figure 2:**
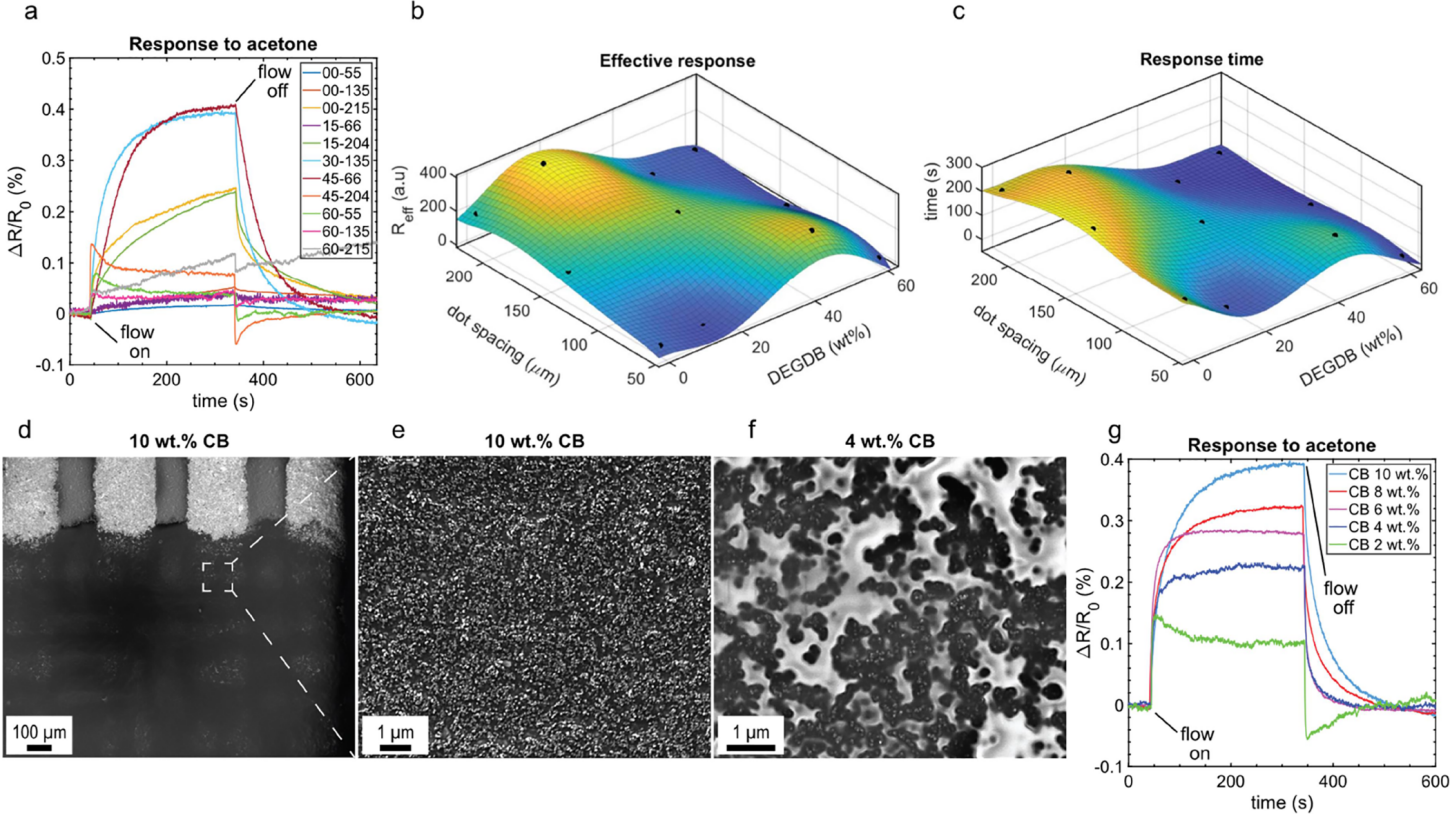
Optimizing the print parameters and ink formulation. (a) Dynamic exposure of the sensors printed with different combinations of the DEGDB concentrations and dot spacings to a fixed acetone concentration (2%). Each sensor is exposed to acetone for 5 min, followed by 5 min recovery. (b) and (c) The sensors’ effective response and response time upon exposure to acetone as a function of the dot spacing and DEGDB concentration, respectively. The effective response is calculated by dividing the maximum response by the standard deviation of the baseline. The response time corresponds to the time it takes to reach 90% of the maximum response. (d) SEM image of a sensory film with 4 wt % CB in the PS-DEGDB matrix. (e) and (f) SEM images of a printed sensory film containing 10 wt % CB in the PS-DEGDB matrix at different magnifications. (g) Dynamic response of sensors containing different CB concentrations upon exposure to a fixed acetone concentration.

The preliminary experiments to find the optimum combination between the plasticizer concentration and the dot spacing are performed by exposing each sensor to a fixed acetone concentration (0.4% v/v), as shown in Figure S5. The features extracted from the third exposure are used for further analysis to avoid the effect of sensor conditioning, which occurs after the first exposure. The sensors’ dynamic response to the third acetone exposure normalized by the baseline resistance is shown in Figure 2a. From the dynamic response, we can deduce that nonplasticized composites show a slow response, as expected. Whereas the response time significantly decreases in sensors containing 30 and 45 wt % DEGDB. Increasing the DEGBD concentration to 60 wt % drops the response time to ∼1 s; however, it also affects the sensor response magnitude and reduces the sensitivity to acetone.

The response magnitude, effective response, and response time are extracted from the dynamic sensor response. The maximum response magnitude (*R*_max_) is calculated based on eq 1, where Δ*R* is the change in the baseline resistance after 5 min of exposure to acetone and *R*_0_ is the baseline resistance at the sensor operation temperature (27 °C). The effective response (*R*_eff_) is calculated from eq 2, where δ is the standard deviation of the baseline resistance. Finally, the response time τ is calculated by fitting a double exponent equation, eq 3, to the response curve, considering the time it takes to reach 90% of the *R*_max_. In eq 3, *R*_t_ is the sensor response at time *t*, *a* and *b* are constants related to the response magnitude and τ_1_and τ_2_ are the time constants.
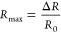
1
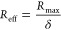
2
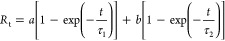
3

The effective response and response time are the main features used to find the optimum plasticizer and dot spacing combination. The response surface plots are shown in Figure 2b,c. The effective response shows two local maxima for sensors 15-204 and 45-66. In sensor 15-204, the large *R*_eff_ is due to its low baseline noise, even though its *R*_max_ is approximately half of the 45-66. Despite the larger *R*_eff_ of 15-204 and 45-66 compared to other sensors, they show a relatively slow response, approximately 3 and 5 min, and a slow and incomplete recovery. This is due to a low degree of plasticization in 15-204 and a relatively large film thickness (∼20 μm) in 45-66. On the other hand, the sensor 30-135, located at the center of the DoE space, shows a comparable *R*_eff_ to 45-66 and 15-204, while having a response time of ∼1 min.

Moreover, due to a low degree of plasticization in 15-204, the sensor recovery is incomplete — due to incomplete desorption of analyte from the polymer — resulting in the baseline drift over time, as seen in Figure S5. On the other hand, due to the excessive plasticization of 45-66, which results in a high polymer chain mobility, the sensor is prone to drift over time. Therefore, sensor 30-135 seems to offer an acceptable trade-off between various parameters, including sensitivity, response time, and stability.

The performance improvement of 30-135 compared to the nonplasticized sensor (00-135) is illustrated by computing the equilibration time calculated based on eq 3 and shown in Figure S6. It is estimated that it takes approximately 1 h for the nonplasticized sensor to reach the steady-state response, whereas reaching equilibrium takes less than 2 min for the sensor 30-135. Furthermore, the response is followed by an incomplete recovery in 00-135, less than 25% of the response. Whereas 30-135 is fully recovered in approximately 2 min. This observation further illustrates the significant improvement of the sensor dynamic by adding an optimum plasticizer concentration.

Considering the discussion above, sensor 30-135, i.e., the composite containing 30 wt % DEGDB and printed with 135 μm dot spacing, is selected for further investigations.

### Effect of the CB Concentration

Another parameter influencing the sensor response is the CB concentration in the composite. Previous studies show that, in polymer composites, at a low mass fraction of CB when approaching the percolation threshold, the sensor becomes more sensitive to VOCs; however, its repeatability deteriorates, and the response becomes nonlinear as a function of the analyte concentration. On the other hand, the sensor shows a lower sensitivity but a linear and repeatable response at relatively high CB mass fractions.^20^ Therefore, the experiments presented in this section aimed to study the effect of CB mass fraction on the performance of sensors printed with 135 μm dot spacing and containing 30 wt % DEGDB.

It is observed that decreasing the CB concentration results in a significant change in the film morphology. The inks containing 6 wt % or lower amounts of CB (in their dry form) result in a nonuniform film morphology shown in Figure S7. Such behavior is linked to the prolonged drying (solidification) time of the sensory film at low CB mass fractions. In inks containing 10 and 8 wt %, CB, droplets dry soon after they land on the substrate, preventing large liquid bead formation and nonuniform drying. However, decreasing the CB mass loading increases the drying time and leads to the formation of large liquid beads on the substrate, which dry unevenly, resulting in a nonuniform film morphology.

Additionally, reducing the CB weight fraction affects the composite’s microstructure. As seen in the SEM images in Figure 2d–f, at 10 wt % loadings, CB particles are uniformly dispersed inside the polymer matrix. However, segregation between CB and the organic matrix is observed by lowering the CB weight fraction to 8 wt %. The segregation becomes more evident by further decreasing the CB concentration to 2 wt %, as shown in Figure S8 b–f.

At 10 wt % CB loading, the CB concentration is presumably above the so-called mechanical percolation threshold, as is evident from the highly interconnected network of CB particles seen in Figure 2b. At this regime, the highly interconnected network of CB particles hinders the mobility of the organic matrix, resulting in a seemingly homogeneous composite matrix at the micrometer scale. However, by decreasing the CB concentration, the mobility of the organic matrix increases since its *T*_g_ is below the room temperature (0 °C at 30 wt % DEGDB). Moreover, free spaces between the CB aggregates begin to appear by decreasing the CB concentration, which is eventually occupied by the organic matrix. The tendency of CB particles to aggregate and segregate from the organic matrix is linked to their interactions with the organic matrix. To tune the dispersion/aggregation behavior of the CB in the PS-DEGDB matrix, one can modify the surface chemistry of the CB particles; however, this was out of the scope of the current work.

Subsequently, the sensory films with different CB loadings are exposed to 0.4% v/v acetone to investigate their sensing performance. It is observed that the response magnitude decreases gradually by reducing the CB concentration, as shown in Figure 2g. This behavior is in contrast with previous observations in nonplasticized PS-CB composites, where reducing CB concentration increased the response magnitude^19^ and can be explained considering the microstructural changes of the composite when reducing its CB concentration. The nonuniform distribution of CB inside the composite matrix at low CB loadings creates CB aggregates with an effective CB concentration higher than the nominal value and DEGDB-rich regions, which are nonconductive. In such composites, the effective sensing area is the CB-rich regions, where due to a high CB concentration, the sensitivity is reduced. The results obtained here point out that the ink composition with 10 wt % CB results in better uniformity of the film morphology and microstructure and higher sensitivity to the target analyte than inks containing lower CB loadings; Hence, the composition with 10 wt % CB was used for further experiments where the selectivity and selectivity of the sensors were studied.

### Selectivity and Sensitivity

Based on the results discussed above, the composition containing 10 wt % CB, 30 wt % DEGDB and printed with 135 μm dot spacing was used for further characterization, including assessing the sensor sensitivity and its concentration dependence upon exposure to various analytes. For these measurements, three sensors were fabricated and exposed to varying concentrations of water, ethanol, 2-propanol, acetone, heptane, benzene, ethylbenzene, and toluene.

**Figure 3:**
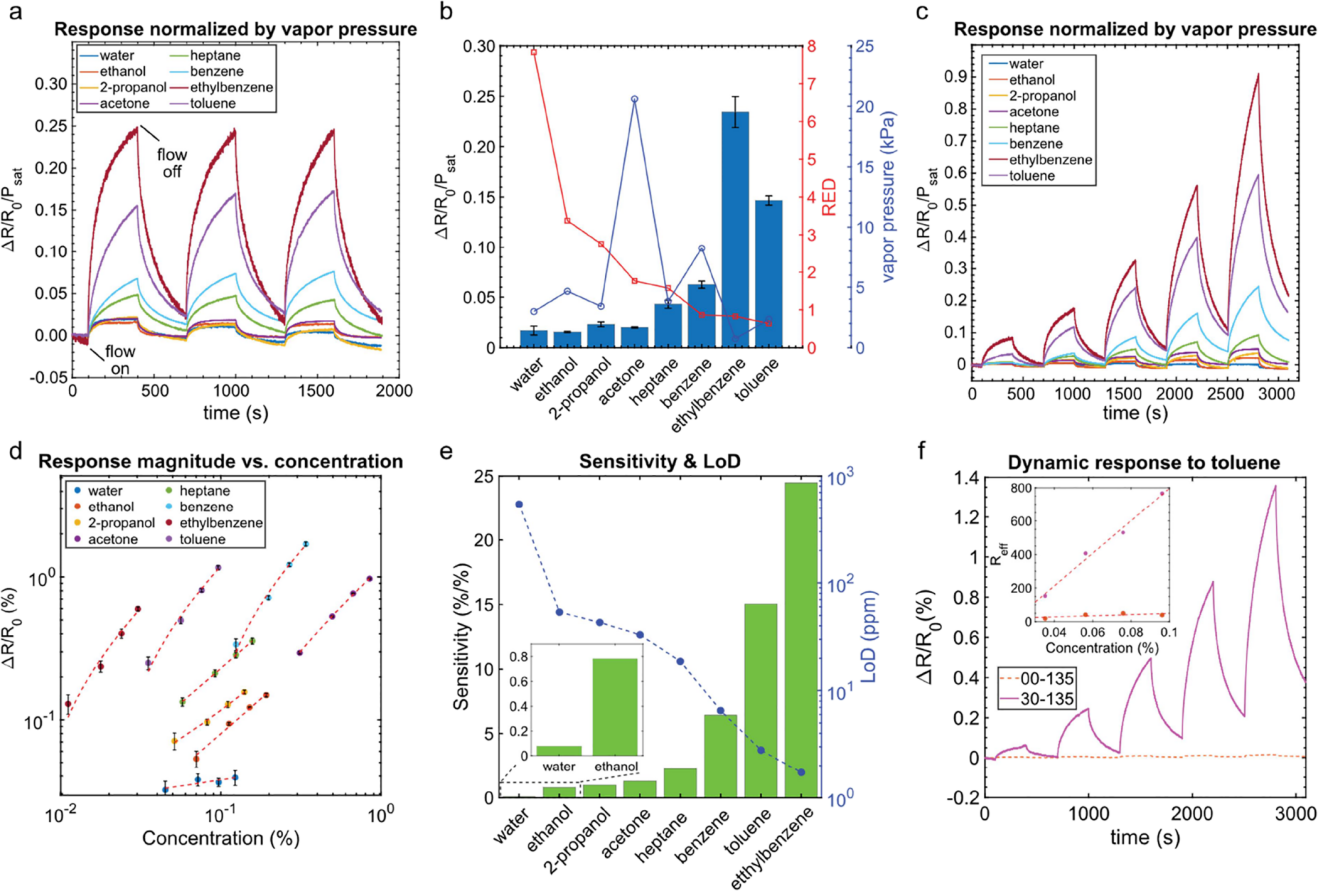
Sensitivity and selectivity of the sensor 30-135 upon exposure to different analytes. (a) Dynamic exposure of the sensor to a fixed concentration of the target analytes. The sensor response is normalized by the baseline resistance and the analyte’s vapor pressure to compare the sensor response directly. (b) Average maximum response of the sensor is calculated from three exposures to each analyte as a function of the RED number and the vapor pressure. (c) Dynamic exposure of the sensor to analytes at different concentrations. The analyte concentration gradually increases in each exposure by increasing the sample flow, dry air saturated with the analyte, from 20 to 100 mL/min with 20 mL/min steps. (d) Average maximum response of three sensors to different concentrations of each analyte, extracted from the dynamic response. The error bars indicate the standard deviation for the average value. (e) Sensor’s sensitivity and LoD toward different analytes, calculated from the response curve vs the analyte concentration. (f) Comparing the dynamic and the effective (inset) response of 00-135 and 30-135 upon exposure to toluene. It is observed that the effective sensitivity of 30-135 is increased by more than 25-fold compared to 00-135 after adding 30 wt % DEGDB.

Figure 3a shows the dynamic response of a representative sensor upon repeated exposure to a fixed concentration of each analyte. The dynamic response is normalized by the sensor baseline resistance and analyte vapor pressure for a direct comparison. The analytes’ vapor pressures are calculated based on Antoine’s equation at the bubbler’s temperature shown in Table S3. It is observed that the sensor has the highest affinity for aromatic compounds, i.e., benzene, toluene, and ethylbenzene. On the other hand, the sensor affinity is reduced toward heptane, a nonpolar linear hydrocarbon. Moreover, the response magnitude decreases further upon exposure to polar aprotic (acetone) and polar protic (ethanol and 2-propanol) analytes. Finally, as expected, the sensor shows a negligible response to water.

The analyte-composite interaction is explained considering two main factors: the solubility of analytes in the polymer matrix and the analytes’ vapor pressure. The solubility of each analyte in the polymer matrix is estimated considering the HSP distance between each polymer-analyte pair using eq S1. The relative energy difference (RED) is then calculated using eq S2. The HSP values of the tested analytes are shown in Table S4, and the calculated HSP distance and RED numbers are shown in Table S5. The RED number of 1 or lower indicates the solubility of an analyte inside the PS matrix, and a RED number slightly larger than 1 indicates that the analyte may cause the polymer to swell. As the RED number increases further, the analyte interaction with the polymer is expected to be minimized.

Figure 3b shows the sensor response magnitude normalized by the analyte vapor pressure versus the RED number. The general trend indicates that as the RED number decreases, the response magnitude increases. The deviation from this trend when comparing the response to acetone and 2-propanol, or benzene, toluene, and ethylbenzene, is linked to the analyte vapor pressure. The partition coefficient of analytes with a low vapor pressure (i.e., high boiling point) is higher than those with high vapor pressure (i.e., low boiling point); This means that at a fixed analyte concentration in the vapor phase, the equilibrium analyte concentration in the polymer phase is higher for analytes with lower vapor pressure. For instance, even though benzene and ethylbenzene have close RED numbers — 0.9 and 0.8, respectively — the sensor shows approximately four times larger response to ethylbenzene due to its lower vapor pressure.

Finally, the concentration dependence of the sensor response is studied by exposure to different analyte concentrations. The normalized dynamic response is shown in Figure 3c. As expected, the sensor response magnitude increases as the analyte concentration. Moreover, as described before, the sensor shows a high affinity to nonpolar aromatic compounds, i.e., benzene, ethylbenzene, and toluene. However, the sensor’s high affinity to BTEX results in its partial recovery and, subsequently, drift from the baseline. The drift becomes more evident as the analyte concentration increases and is more prominent after exposure to ethylbenzene due to its low vapor pressure.

Subsequently, the sensitivity is calculated by plotting the response magnitude as a function of the analyte concentration. The analytes’ volume concentration is calculated based on eq S3. Figure 3d shows the response magnitude as a function of the concentrations, where each data point represents the average response obtained by measuring three sensors and the error bar corresponds to the standard deviation from the average values.

The sensors show a linear dependency on the analyte concentration in the measurement range. The slope of the response curves indicates the sensitivity of each analyte. The dashed lines show the linear fit to the sensor response from which the sensitivities are calculated and plotted for each analyte, as shown in Figure 3e. The sensitivity to different analytes follows the same trend as discussed before and is determined based on the solubility parameters and analytes’ vapor pressures. For instance, as shown in Figure 3e, the sensor shows the highest sensitivity to ethylbenzene due to its low vapor pressure and high solubility in the PS matrix.

An advantage of using a PS-based polymer composite is its negligible sensitivity to water due to the insolubility of water in either PS or DEGDB. The negligible sensitivity to water is highly desirable since humidity often interferes with detecting the analytes of interest in chemical sensors. Therefore, such a detector can be used for the selective detection of VOCs with a high affinity toward aromatic hydrocarbons, including BTEX compounds.

Another important metric for the chemical sensors is their LoD. The sensor’s LoD to each analyte is calculated from the sensitivity values considering that the minimum detectable signal is three times the baseline noise. It should be noted that the measurement is done over one full power line cycle, corresponding to an integration time of ∼20 ms and yielding a good degree of noise rejection. Presuming that the sensor response remains linear, the LoD at 27 °C is below 10 ppm for benzene, toluene, and ethylbenzene and below 100 ppm for the polar protic and aprotic analytes (ethanol, acetone, and 2-propanol). As expected, when HSPs are close, the LoD decreases for analytes with a lower vapor pressure. Table S7 of the Supporting Information compares the key features of various chemiresistive sensors with the plasticized polymer composite studied in this work.

Finally, to highlight the effect of plasticizers on the sensors’ sensitivity to BTEX compounds, the dynamic and effective responses of sensors 00-135 and 30-135 are directly compared by exposing the sensor to the different toluene concentrations (Figure 3f). It is shown that the sensor’s effective sensitivity (*R*_eff_) is increased by more than 25 times when adding 30 wt % plasticizer to the composition. Such a significant increase in sensitivity, on the one hand, is linked to the solubility of toluene (as well as other BTEX compounds) in both PS and DEGDB, and, on the other hand, to the improved dynamics of the sensor response induced by the plasticizer, allowing faster equilibration.

## Conclusions

The effect of a plasticizer (DEGDB) on the performance of VOC sensors composed of a polymer (PS) matrix and CB fillers is studied. The sensory material is deposited via drop-on-demand inkjet printing, allowing for a high degree of control over the amount of the deposited material and the film morphology. The primary composition and fabrication parameters, i.e., DEGDB concentration and dot spacing) are studied via a DoE method. It is shown that optimum sensor performance and fast response while maintaining high sensitivity are obtained at a specific combination of dot spacing (135 μm) and plasticizer concentration (30 wt %).

Additionally, the effect of CB concentration on the sensor performance is studied, showing that sensors containing 10 wt % CB would result in optimum performance. We also observed that reducing the CB concentration does not improve the sensitivity, and in contrast, it results in the composite phase separation and significantly deteriorates the sensor response.

Furthermore, the sensitivity and selectivity of the optimum sensor upon exposure to various analytes are investigated. It is observed that the PS-DEGDB-CB sensor is highly sensitive and selective toward aromatic hydrocarbons such as benzene, toluene, and ethylbenzene, with a sub-10 ppm LoD while showing a low sensitivity toward humidity. The optimized sensor shows approximately 25 times better effective sensitivity than the PS-CB composite printed with the same parameters while operating at near room temperature (27 °C), providing a sensitive and cost-effective sensing element for detecting highly toxic BTEX compounds. Even though the LoD of the sensors presented here do not yet comply with the specifications of the most demanding applications such as environmental monitoring or biomarker detection that require sub-ppm LoD, further improvement in terms of the design, signal processing, and analyte collection system can now be undertaken to reduce the LoD by several orders of magnitude. This can be achieved for instance by well-known methods such as introducing a gas preconcentrator to the sensor design^20^ and/or utilizing impedance measurement techniques.^21^

Finally, such plasticized polymer composites have potential applications for flexible and stretchable electronic devices. This is due to their low fabrication and operation temperature and printability, as well as mechanical and chemical compatibility with various flexible substrates such as polyimide, polyethylene naphthalate, and polyethylene terephthalate. The proposed sensing systems also comply with sustainability aspects such as reduced waste of materials due to the digital printing approach and on-demand manufacturing.

## Supporting Information Available

Additional experimental details, including ink formulation, TGA of the polymer and plasticizer, optical and SEM images of printed sensory films, film thickness measurement, dynamic sensor response to acetone, solubility calculations, and table of comparison to state-of-the-art chemiresistive sensors (PDF)
