# Supplementary material for: Near-Room-Temperature Detection of Aromatic Compounds with Inkjet-Printed Plasticized Polymer Composites
Source: ACS Sens. 2024 Mar 13;9(3):1382–90. doi: 10.1021/acssensors.3c02406 (PMC10964229; doi:10.1021/acssensors.3c02406)
Supplement: Supplementary file 1 — se3c02406_si_001.pdf [file se3c02406_si_001.pdf]

Supporting information

Near-room-temperature detection of aromatic compounds with inkjet-printed plasticized polymer composites

M. M. Kiaee[a#], T. Maeder[a$], J. Brugger[a*]

[a]*Microsystem Laboratory, École Polytechnique Fédérale de Lausanne (EPFL), CH-1015 Lausanne, Switzerland*

* Email: juergen.brugger@epfl.ch

Ink formulation

Formulated inks based on the Doehlert method are shown in Table S1. The inks contain a fixed CB loading, while the plasticizer concentration is varied. The inkjet inks were formulated by dissolving PS, DEGDB, and subsequently dispersing CB in a mixture of PGMEA (70 wt%) and DPGEMA (30 wt%). In the inkjet inks studied in this work, the sensing materials (i.e., PS, DPGMEA, and CB) constituted 12 wt% of the inks by weight. The ratio between the two solvents and the total weight fraction of the sensing material was selected according to our previous studies. Moreover, the composition of the composites used to study the CB effect is shown in Table S2.

Table S1: The nominal weight and volume fractions of PS, DEGDB, and CB in dry composites. The sensors printed from the following composites were used to find the optimum DEGDB concentration.

**Ink**	**PS**		**DEGDB**		**CB**	
	**wt%**	**vol%**	**wt%**	**vol%**	**wt%**	**vol%**
**DEGDB-1**	90	94	0	0	10	6
**DEGDB-2**	77	81	14	13	10	6
**DEGDB-3**	63	68	27	26	10	6
**DEGDB-4**	50	54	41	39	10	6
**DEGDB-5**	36	40	54	53	10	6

Table S2: The nominal weight and volume fractions PS, DEGDB, and CB in dry composites. The sensors printed from the following composites were used to study the effect of CB loading on the sensor response.

**Ink**	**PS**		**DEGDB**		**CB**	
	wt%	vol%	wt%	vol%	wt%	vol%
**CB-1**	63.0	67.8	27	25.9	10	6.3

**CB-2**	64.4	68.8	27.6	26.2	8	5.0
**CB-3**	65.8	69.7	28.2	26.6	6	3.7
**CB-4**	67.2	70.6	28.8	26.9	4	2.5
**CB-5**	68.6	71.5	29.4	27.3	2	1.2

Material characterization

Figure S1 shows the thermogravimetric analysis (TGA) of PS and DEGDB under synthetic dry air flow.

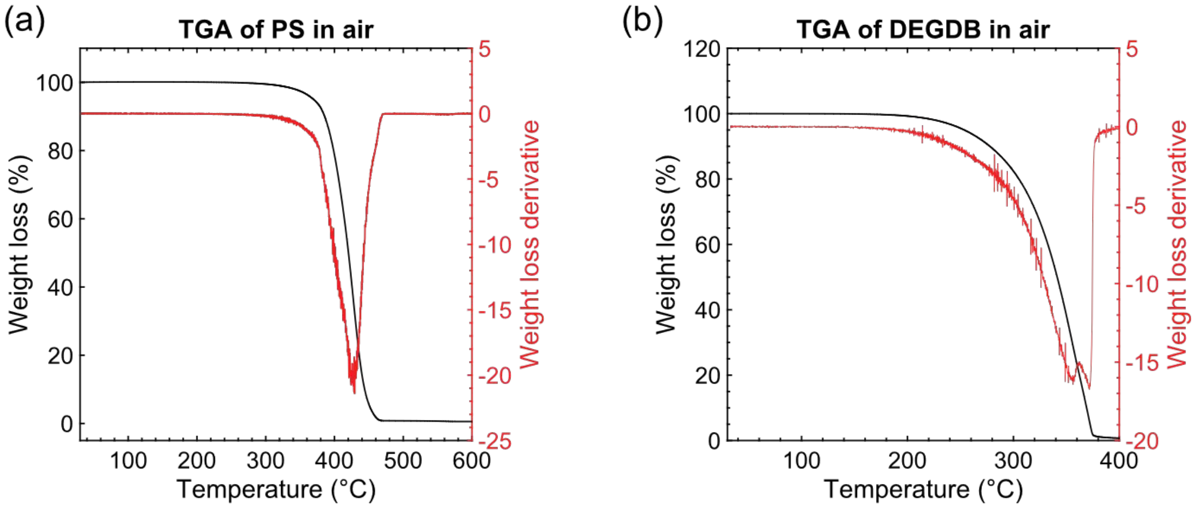

Figure S1: Thermogravimetric analysis (TGA) of (a) polystyrene and (b) DEGDB. The TGA is performed under a synthetic dry air flow. The temperature range is set from 30 to 400 °C for DEGDB and 30 to 700°C for PS characterization, with heating rates of 10°C/min. Based on the TGA results, PS and DEGDB are stable up to ~350 and ~200°C, respectively.

Sensor fabrication and characterization

Figure S2 shows images of the inkjet-printed sensory films taken by a stereomicroscope and an optical microscope at different magnifications.

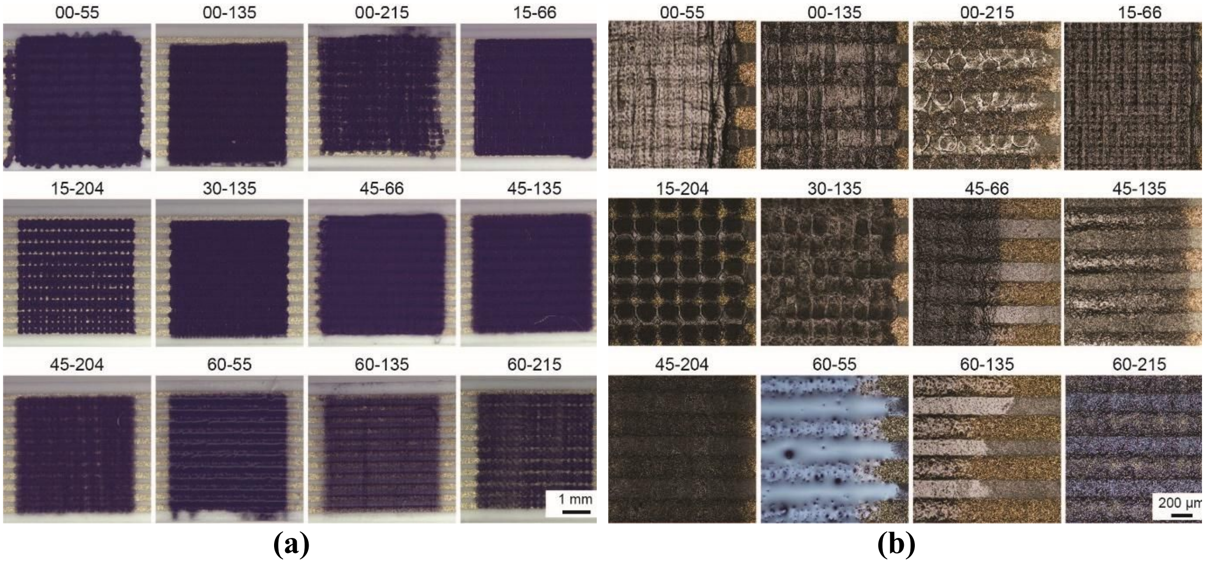

*Figure S2: The morphology of the printed sensory films **(a)** Stereomicroscope and **(b)** optical microscope images of the printed sensory films. The numbers above each image indicate the combination of plasticizer concentration (wt%) and dot spacing (µm).*

Figure S3 shows the surface profiles of inkjet-printed sensors measured by a Dektak mechanical profilometer.

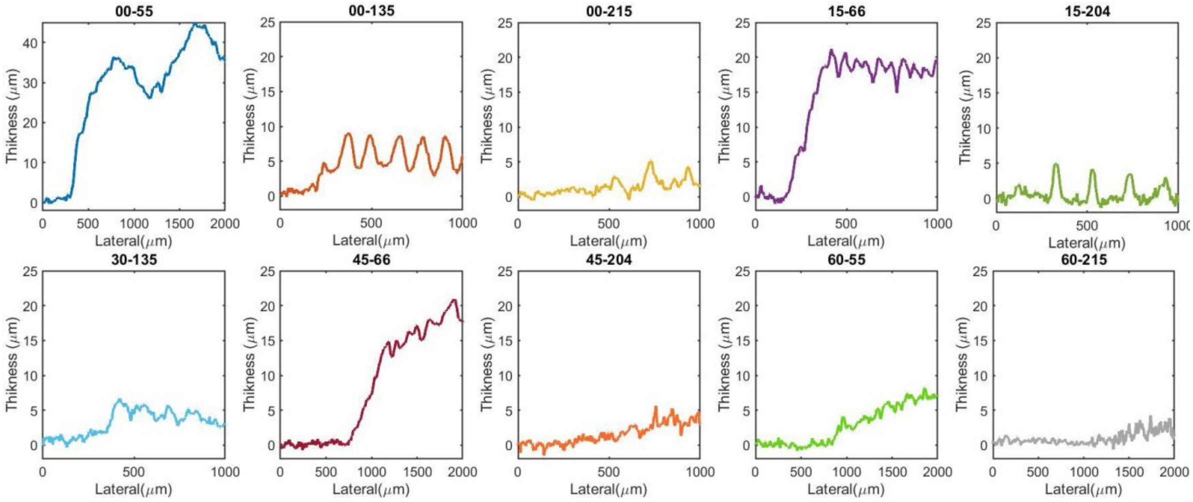

Figure S3: Film thickness measured with a Bruker Dektak mechanical profilometer. The thickness of the sensory films printed with a dot spacing larger than 200 µm becomes comparable with alumina surface roughness, making it difficult to measure the thickness.

Figure S4 shows SEM images of the inkjet-printed sensory films containing different concentrations of DEGDB. The dark zones are CB-rich, and the bright ones contain little or no CB.

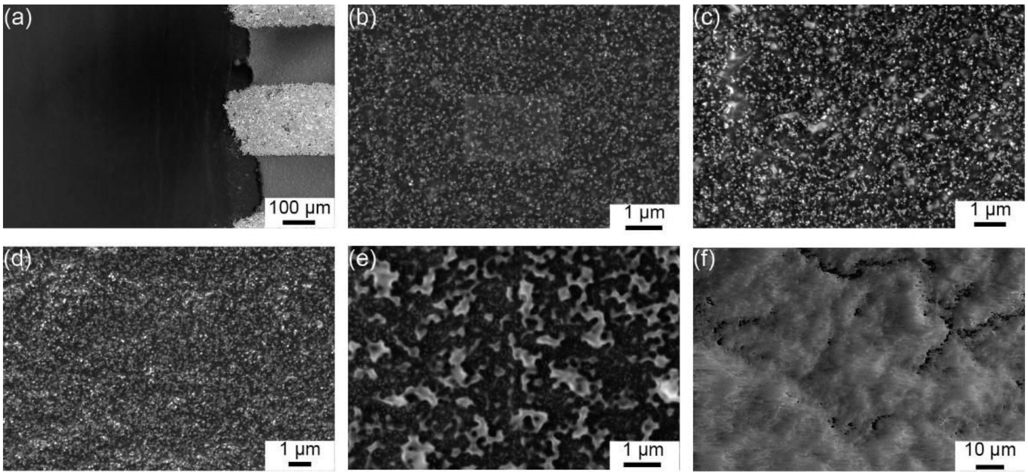

Figure S4: SEM images of sensory films containing different DEGDB concentrations. (a) and (b) the sensory film with no plasticizer at different magnifications. (c), (d), (e), (f): sensory films containing 15, 30, 45, and 60 wt% DEGDB, respectively.

Figure S5 shows the sensors' dynamic responses after exposure to a contact acetone concentration.

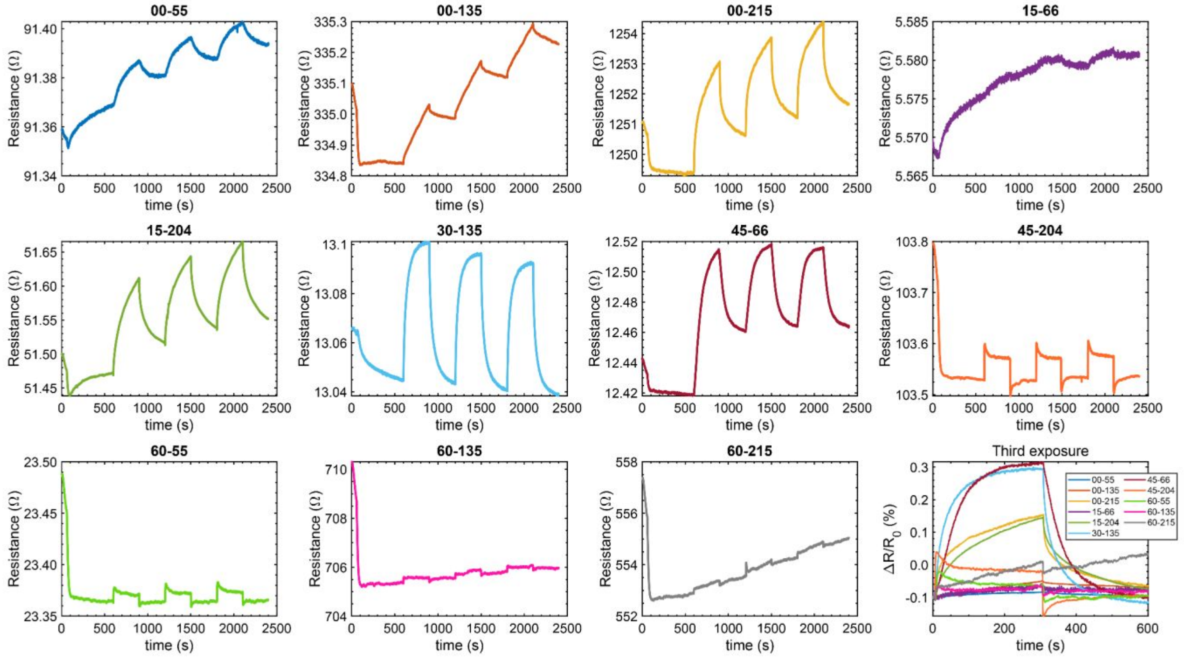

Figure S5: Effect of sensor exposure to acetone. Each sensor is exposed to 0.4 % acetone three times during 5 min exposure and 5 min recovery cycles. The sensors are measured at 27°C. The initial drop of the sensor resistance results from increasing the temperature from the room temperature to 27°C. Subsequently, exposing the sensors to acetone results in increasing the baseline resistance. The last figure shows the normalized sensor response during the third exposure.

Figure S6 shows a comparison between the dynamic response of non-plasticized and plasticized composites.

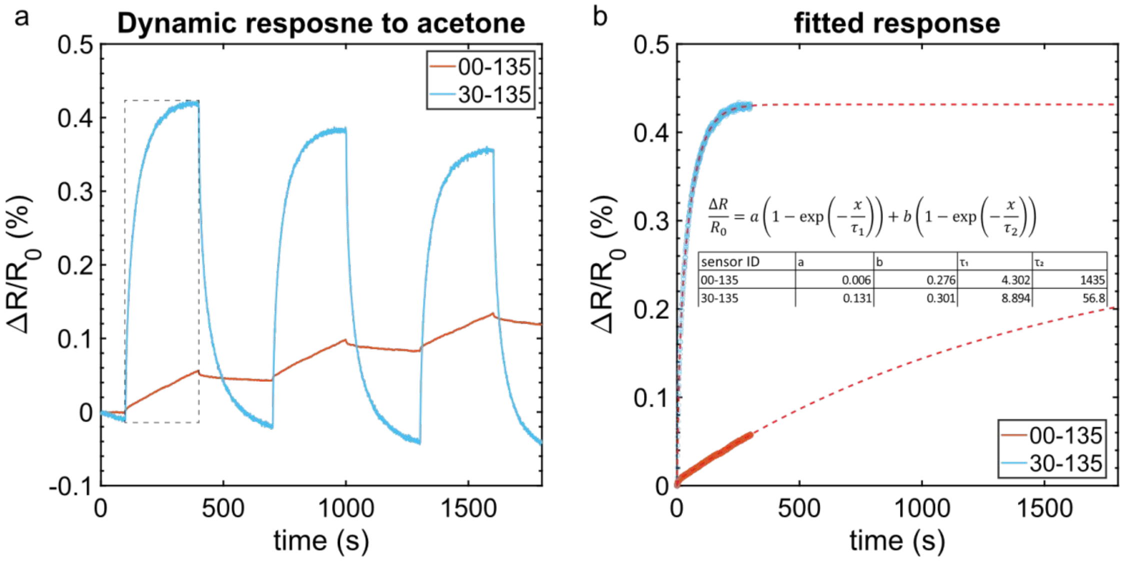

Figure S6: Direct comparison between the dynamic response of PS-CB composites with (30 wt%) and without plasticizer. (a) Dynamic responses upon exposure to 0.4% acetone. The dotted rectangle shows the portion of the sensor response used for fitting. (b) fitting the dynamic response by a double exponent equation to estimate the time for each sensor to reach its equilibrium. The equation and fitted parameters are shown in the inset of the figure. Extrapolating the data shows that it takes the sensor 00-135 approximately 60 minutes to equilibrate, whereas the sensor 30-135 reaches the steady-state in less than 2 minutes. Moreover, 30-135 fully recovers after a few minutes, whereas 00-135 only partially recovers, resulting in a significant baseline drift after each exposure to acetone.

Figure S7 shows he micrographs of the sensory films containing different CB concentrations are shown in figure S8 below.

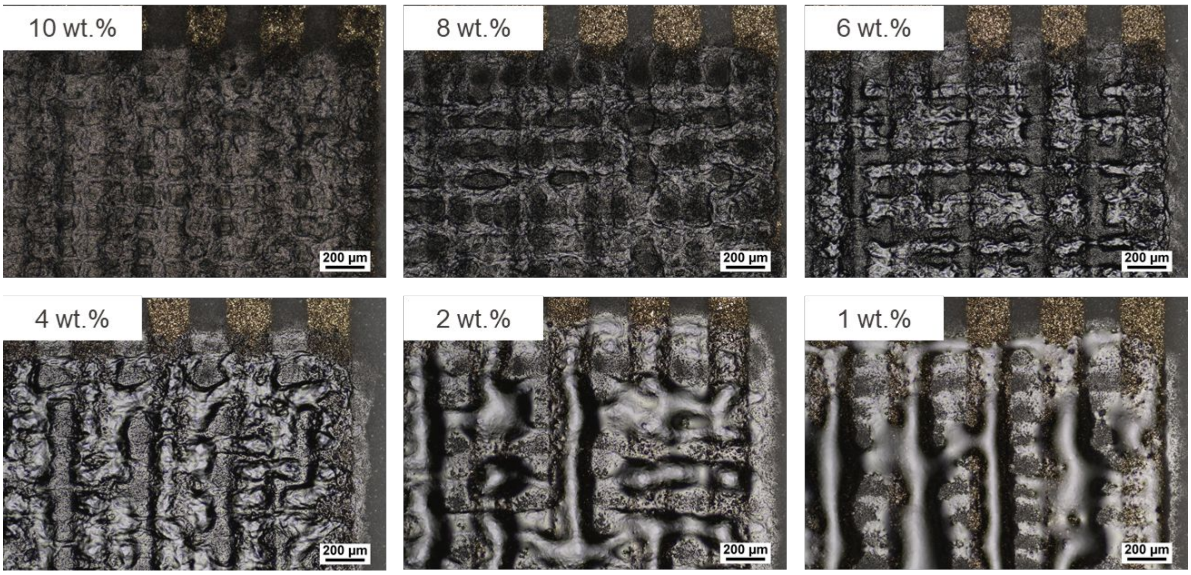

Figure S7: Optical microscope images of the printed sensory film containing different CB concentrations. The sensory films contain (a) 10 wt%, (b) 8 wt%, (c) 6 wt%, (d) 4 wt%, (e) 2 wt%, and (f) 1 wt% CB. It is observed that the film morphology and uniformity are affected significantly by decreasing the CB concentration

Figure S8 shows SEM images of sensory films containing different CB concentrations. The films were prepared with 135 μm dot spacing and 30 wt% DEGDB in the organic matrix.

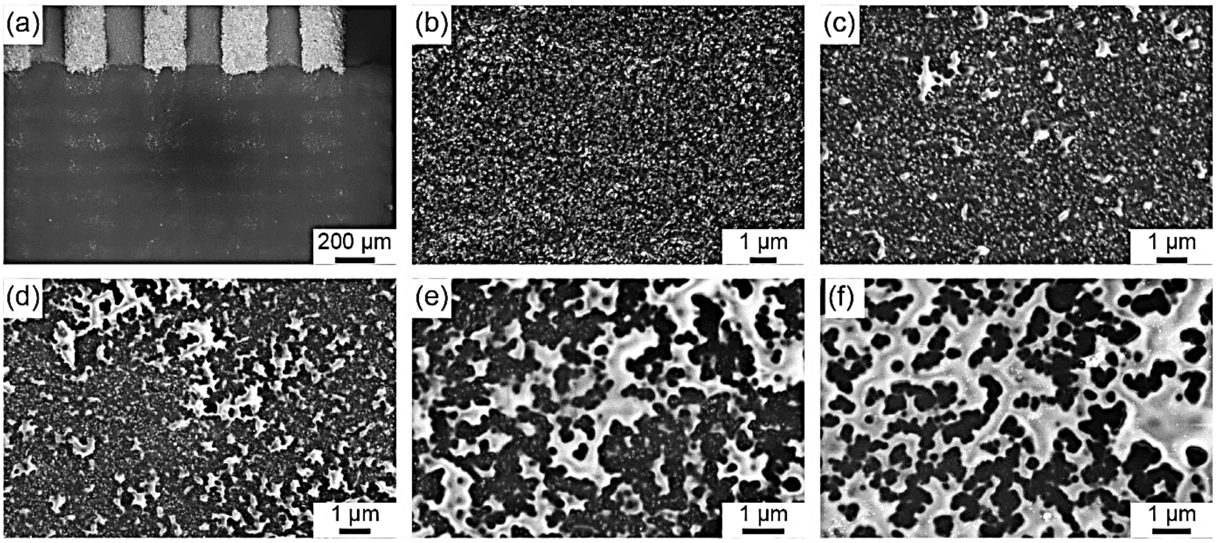

Figure S8: SEM images of sensory films containing (a) and (b) 10 wt%, (c) 8 wt%, (d) 6 wt%, (e) 4 wt%, and (f) 2 wt% CB. The microstructure of the sensory film changes by decreasing the CB concentration, becoming visibly coarser and less homogeneous when the CB concentration is decreased.

Table S3 shows the vapor pressures of the used analytes calculated using the Antoine equation.

Table S3: Vapor pressure of target analytes calculated based on the Antoine equation (log(p)=A-B/(C+T)) [1]

Analyte	A	B	C	T (°C)	P (kPa)
**Water**				24	2.985
**Ethanol**	8.32109	1718.1	237.52	16	4.667
**2-propanol**	8.11778	1580.92	219.61	16	3.410
**Acetone**	7.11714	1210.595	229.664	16	20.615
**Heptane**	6.89677	1264.9	216.54	16	3.821
**Benzene**	6.90565	1211.033	220.79	16	8.245
**Ethylbenzene**	6.95719	1424.255	213.21	16	0.738
**Toluene**	6.95464	1344.8	219.48	16	2.337

Equations S1 and S2 below are used to calculate the HSP distance and the RED number.

$$R_a^2 = 4(\delta_{d1} - \delta_{d2})^2 + (\delta_{p1} - \delta_{p2})^2 + (\delta_{h1} - \delta_{h2})^2 \qquad \textit{Eq. S1}$$

$$RED = \frac{R_a}{R_0} \qquad \textit{Eq. S2}$$

Table S4 below shows the HSPs of the analytes used for sensor characterization.

Table S4: Hansen Solubility Parameters of target analytes and polystyrene used to calculate the HSP distance. [2]

Analyte/polymer	Polar ($MPa^{1/2}$)	Dispersion ($MPa^{1/2}$)	Hydrogen bonding ($MPa^{1/2}$)
**Toluene**	18.0	1.4	2.0
**Benzene**	18.4	0.0	2.0
**Ethylbenzene**	17.8	0.6	1.4
**Xylene**	17.6	1.0	3.1
**Heptane**	15.3	0.0	0.0
**Acetone**	15.5	10.4	7
**Ethanol**	15.8	8.8	19.4
**2-propanol**	15.8	6.1	16.4
**water**	15.5	16	42.3
**Polystyrene**	18.5	4.5	2.9

Table S5 below shows the calculated HSP and RED numbers.

Table S5: HSP distance (R_a) and RED number calculated from the HSP values of Table S4. The polystyrene solubility radius (R_0) is considered 5.3 MPa$^{1/2}$ to calculate the RED number.

analyte-polymer pair	HSP distance	RED
**PS-toluene**	3.4	0.6
**PS-benzene**	4.6	0.9
**PS-ethylbenzene**	4.4	0.8
**PS-xylene**	3.9	0.7
**PS-heptane**	8.3	1.6
**PS-acetone**	9.4	1.8
**PS-ethanol**	17.9	3.4
**PS-2 propanol**	14.6	2.8
**PS-water**	41.5	7.8

Table S6 shows the HSPs of PS, DEGDB and PS-DEGDB mixture. The HSPs of DEGDB are calculated using a group contribution method proposed by Stefanis et.al.[2] as we could not find any experimental studies regarding the solubility parameters of DEGDB. The HSPs of the PS-DEGDB mixture are then calculated based on the nominal volume fractions in a mixture containing 72 vol% PS and 28 vol% DEGDB, corresponding to 70 wt% PS and 30 wt% DEGDB in the organic matrix.

Moreover, based on the computed values, the solubility distance and the RED number for the PS-DEGDB pair are 8.5 MPa$^{1/2}$, and 1.6, respectively, indicating that PS and DEGDB are not fully miscible. The solubility limit of PS-DEGDB is presumably linked to higher polarity and hydrogen bonding numbers of DEGDB than PS.

Table S6: HSPs of PS, DEGDB, and PS-DEGDB mixture, The HSPs of the DEGDB are calculated using the group contribution method

Analyte/polymer	Dispersion (MPa$^{1/2}$)	Polar (MPa$^{1/2}$)	Hydrogen bonding (MPa$^{1/2}$)
**PS**	18.5	4.5	2.9
**DEGDB**	20.2	12.2	4.5
**PS-DEGDB**	19.0	6.7	3.3

Figure S9 compares the HSP distance of PS-analyte pairs versus that of the PS-DEGDB-analyte pairs. It is clear that due to the higher polarity of the PS-DEGDB mixture, the solubility distance to polar compounds decreases, whereas the distance to the non-polar compounds increases. In other words, the composite containing the PS-DEGDB mixture becomes slightly more sensitive to polar compounds than the sensor containing only PS. However, the general trend does not change, indicating that the solubility distance and RED numbers considering only PS can explain the sensors' behavior.

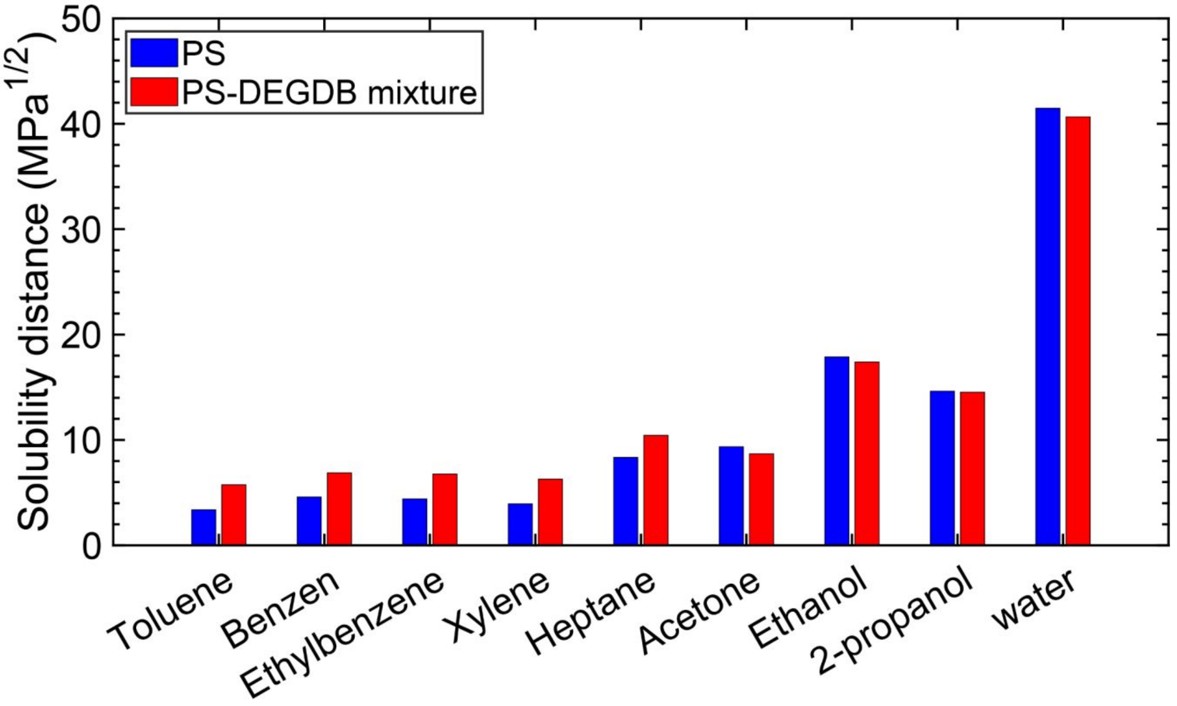

Figure S9: A comparison between solubility distance of PS and PS-DEGDB mixture with various analytes. Due to the higher polarity of the PS-DEGDB mixture, it has a lower solubility distance to polar analytes and a larger distance to non-polar analytes compared to PS. However, the general trend, regarding the affinity of the sensor to the target analytes does not show a considerable change.

Equation S3 below is used to calculate the analyte concentration.

$$c(\%) = \frac{F_a}{F_a + F_b} . \frac{\rho_i}{P} \qquad \textit{Eq. S3}$$

Table S7, below, shows a comparison between the key features of chemiresistive sensors based on their sensing materials, including metal oxides (MOX), Graphene, transitional metal dichalcogenides (TMDCs), carbon nanotube (CNT), MXenes, metal-organic frameworks (MOFs), polymers and polymer composites. The selected sensors show the performance upon exposure to acetone, ethanol, and aromatic hydrocarbons for comparison with the sensors fabricated in this work.

The main interest in conductive polymers and polymer composites for VOC sensing is their operation at room temperature, resulting in lower power consumption than MOXs. Moreover, polymers are inherently selective towards various VOCs, considering their chemical structure. The affinity of a polymer to VOCs can be easily estimated via the application of Hansen Solubility Parameters, which allows for predicting the sensor's behavior. Moreover, polymers can be readily processed and adapted to various additive fabrication methods, which is convenient for depositing sensing materials. However, as explained in the manuscript, some polymers, such as PS, offer interesting sensing properties for detecting VOCs, but they are limited by the kinetics of the sensor response. Here it is shown that additives such as plasticizers can significantly improve the polymer's sensing performance without affecting the processability and sensitivity of PS-based composite. As shown in the table, the performance of PS-DEGDB-CB is comparable to examples of room temperature MOXs, 2D materials, CNT, and polymer composites. However, it lags behind more advanced materials such as hetero MOXs, operating at elevated temperatures, MOF-MOX hybrids, and MXenes. Nevertheless, PS-DEGDB-CB still offers comparable performance for detecting aromatic compounds at/near room temperature. Moreover, the composite formulated here is a suitable material for stretchable and flexible sensor applications.

Table S7: Comparing key features of chemiresistive sensors composed of various sensing materials

Material class	***Sensing material***	***Analyte***	***Operating condition***	***LoD***	***Response time***	***Ref.***
MOX (nanosheet)	*SnO_2 nanosheets*	*acetone*	*Dry air 300°C*	*5 ppm***	*1s*	*[3]*
Hetero MOX	*Fe_2O_3/NiO*	*toluene*	*300°C*	*18.68 to 100 ppm*	*1s*	*[4]*

Hetero MOX	*NiO–SnO_2*	*toluene*	*330°C*	*11 to 50 ppm*	*2s*	*[5]*
Hetero MOX	*Au–SnO_2*	*benzene toluene p-xylene ethanol*	*350°C*	-	-	*[6]*
Hetero MOX (nanowire)	*V_2O_5 nanowire decorated with SnO_2*	*ethanol*	*Dry air RT*	*10 ppm***	-	*[7]*
Hetero MOX (core-shell)	*Co_3O_4–SnO_2*	*xylene methylbe-nzene*	*275°C 300°C*	-	-	*[8]*
Graphene (composite)	*Graphene SnO_2 hybrid composite*	*acetone*	*Dry air RT*	*<10 pm*	*107 s*	*[9]*
Graphene (Hybrid)	*3D graphene-CNT decorated by TiO_2*	*toluene*	*Humid air (56% RH) 26°C*	*50 ppm***	*<10s*	*[10]*
TMDC	*MoS_2*	*acetone ethanol hexane toluene*	*N_2*	*<1 ppm* 3 ppm* 3 ppm* 3 ppm**	-	*[11]*
CNT (composite)	*functional cellulose /SWCNT bi-layer*	*Benzene toluene xylene*	*N_2 RT*	*55 ppm* 19 ppm* 14 ppm**	*<10s*	*[12]*
CNT (functionalized)	*PEDOT:PSS-MWCNT*	*ethanol*	*Dry air RT*	*13 ppm**	*13s @ 1000 ppm*	*[13]*
Mxene	*Mo_2CT_x*	*toluene*	*N_2 RT*	*220 ppb*	-	*[14]*
MOF	*ZIF-8-derived PdO-ZnO-SnO_2 nanotubes*	*acetone*	*Humid Air (95% RH) 400 °C*	*100 ppb***	*20s*	*[15]*
Polymer	*polythiophene*	*acetone toluene*	*N_2 RT*	*170 ppm 10 ppm*	*30s*	*[16]*

Polymer composite	*aPVDF-HFP/bC65/CNT*	*acetone*	*N_2 22 °C*	*40 ppm***	*200s*	*[17]*
Polymer composite	*PS-DEGDB-CB*	*toluene*	*Dry air 27°C*	*2 ppm**	*100s*	***this work***

References:

[1] J. Speight, *Lange's Handbook of Chemistry*, Seventeenth Edition. McGraw Hill Higher Education, 2016.

[2] E. Stefanis and C. Panayiotou, 'Prediction of Hansen Solubility Parameters with a New Group-Contribution Method', *Int J Thermophys*, vol. 29, no. 2, pp. 568–585, Apr. 2008, doi: 10.1007/s10765-008-0415-z.

[3] Z. Lou, L. Wang, R. Wang, T. Fei, and T. Zhang, 'Synthesis and ethanol sensing properties of SnO2 nanosheets via a simple hydrothermal route', *Solid-State Electronics*, vol. 76, pp. 91–94, Oct. 2012, doi: 10.1016/j.sse.2012.05.062.

[4] C. Wang *et al.*, 'Hierarchical α-Fe2O3/NiO Composites with a Hollow Structure for a Gas Sensor', *ACS Appl. Mater. Interfaces*, vol. 6, no. 15, pp. 12031–12037, Aug. 2014, doi: 10.1021/am501063z.

[5] L. Liu *et al.*, 'High toluene sensing properties of NiO–SnO2 composite nanofiber sensors operating at 330°C', *Sensors and Actuators B: Chemical*, vol. 160, no. 1, pp. 448–454, Dec. 2011, doi: 10.1016/j.snb.2011.08.007.

[6] Y. K. Moon, S.-Y. Jeong, Y. C. Kang, and J.-H. Lee, 'Metal Oxide Gas Sensors with Au Nanocluster Catalytic Overlayer: Toward Tuning Gas Selectivity and Response Using a Novel Bilayer Sensor Design', *ACS Appl. Mater. Interfaces*, vol. 11, no. 35, pp. 32169–32177, Sep. 2019, doi: 10.1021/acsami.9b11079.

[7] R. Wang *et al.*, 'Enhanced gas sensing properties of V 2 O 5 nanowires decorated with SnO 2 nanoparticles to ethanol at room temperature', *RSC Advances*, vol. 5, no. 51, pp. 41050–41058, 2015, doi: 10.1039/C5RA00530B.

[8] H.-M. Jeong, J.-H. Kim, S.-Y. Jeong, C.-H. Kwak, and J.-H. Lee, 'Co3O4–SnO2 Hollow Heteronanostructures: Facile Control of Gas Selectivity by Compositional Tuning of Sensing Materials via Galvanic Replacement', *ACS Appl. Mater. Interfaces*, vol. 8, no. 12, pp. 7877–7883, Mar. 2016, doi: 10.1021/acsami.6b00216.

[9] D. Zhang, A. Liu, H. Chang, and B. Xia, 'Room-temperature high-performance acetone gas sensor based on hydrothermal synthesized SnO2-reduced graphene oxide hybrid composite', *RSC Adv.*, vol. 5, no. 4, pp. 3016–3022, Dec. 2014, doi: 10.1039/C4RA10942B.

[10] Y. Seekaew, A. Wisitsoraat, D. Phokharatkul, and C. Wongchoosuk, 'Room temperature toluene gas sensor based on TiO2 nanoparticles decorated 3D graphene-carbon nanotube nanostructures', *Sensors and Actuators B: Chemical*, vol. 279, pp. 69–78, Jan. 2019, doi: 10.1016/j.snb.2018.09.095.

[11] J.-S. Kim, H.-W. Yoo, H. O. Choi, and H.-T. Jung, 'Tunable Volatile Organic Compounds Sensor by Using Thiolated Ligand Conjugation on MoS2', *Nano Lett.*, vol. 14, no. 10, pp. 5941–5947, Oct. 2014, doi: 10.1021/nl502906a.

[12] J. Im, E. Sterner, and T. Swager, 'Integrated Gas Sensing System of SWCNT and Cellulose Polymer Concentrator for Benzene, Toluene, and Xylenes', *Sensors*, vol. 16, no. 2, p. 183, Feb. 2016, doi: 10.3390/s16020183.

[13] A. S. Alshammari, M. R. Alenezi, K. T. Lai, and S. R. P. Silva, 'Inkjet printing of polymer functionalized CNT gas sensor with enhanced sensing properties', *Mater. Lett.*, vol. 189, pp. 299–302, Feb. 2017, doi: 10.1016/j.matlet.2016.11.033.

[14] W. Guo *et al.*, 'Selective Toluene Detection with Mo2CTx MXene at Room Temperature', *ACS Appl. Mater. Interfaces*, vol. 12, no. 51, pp. 57218–57227, Dec. 2020, doi: 10.1021/acsami.0c16302.

[15] W.-T. Koo, J.-S. Jang, S.-J. Choi, H.-J. Cho, and I.-D. Kim, 'Metal–Organic Framework Templated Catalysts: Dual Sensitization of PdO–ZnO Composite on Hollow SnO2 Nanotubes for Selective Acetone Sensors', *ACS Appl. Mater. Interfaces*, vol. 9, no. 21, pp. 18069–18077, May 2017, doi: 10.1021/acsami.7b04657.

[16] B. Li *et al.*, 'Inkjet printed chemical sensor array based on polythiophene conductive polymers', *Sensors and Actuators B: Chemical*, vol. 123, no. 2, pp. 651–660, May 2007, doi: 10.1016/j.snb.2006.09.064.

[17] A. Daneshkhah, S. Shrestha, M. Agarwal, and K. Varahramyan, 'Poly(vinylidene fluoride-hexafluoropropylene) composite sensors for volatile organic compounds detection in breath', *Sensors and Actuators B: Chemical*, vol. 221, pp. 635–643, Dec. 2015, doi: 10.1016/j.snb.2015.06.145.
